# The Frequency of Risk Factors for Cleft Lip and Palate in Mexico: A Systematic Review

**DOI:** 10.3390/diagnostics14161753

**Published:** 2024-08-12

**Authors:** Sandra López-Verdín, Judith A. Solorzano-López, Ronell Bologna-Molina, Nelly Molina-Frechero, Omar Tremillo-Maldonado, Victor H. Toral-Rizo, Rogelio González-González

**Affiliations:** 1 Health Science Center, Research Institute of Dentistry, Universidad de Guadalajara, Guadalajara 44100, Mexico; sandra.lverdin@academicos.udg.mx (S.L.-V.); judith.solorzano6225@alumnos.udg.mx (J.A.S.-L.); 2 Molecular Pathology Area, School of Dentistry, Universidad de la República, Montevideo 11400, Uruguay; ronellbologna@odon.edu.uy; 3 Department of Research, School of Dentistry, Universidad Juárez del Estado de Durango, Durango 34070, Mexico; omar.tremillo@ujed.mx; 4 Department of Health Care, Universidad Autónoma Metropolitana Xochimilco, México City 04960, Mexico; nmolina@correo.xoc.uam.mx; 5 School of Dentistry, National Autonomous University of Mexico, Toluca de Lerdo 50000, Mexico; vhtoralr@uaemex.mx

**Keywords:** cleft lip and palate, risk factors, Mexican population, toxic substances

## Abstract

Background: Cleft lip and palate is an anomaly that affects both women and men. It is considered to be among the most frequent congenital abnormalities and is related to modifications in chromosomal DNA and multiple genetic alterations. This anomaly can also be associated with various environmental factors, such as tobacco and alcohol consumption, medication use, and exposure to different environmental and industrial toxic substances. The objective of this study was to document the frequency of risk factors related to cleft lip and palate through a systematic review of Mexican studies. Methods: In this systematic review, a bibliographic search was conducted following PRISMA guidelines in the databases Scielo, ScienceDirect, PubMed, and EBSCO. Keywords related to cleft lip and palate, epidemiology, and risk factors were used. In all, 3 independent reviewers (J.A.S.L., S.L.V., and N.M.F.) selected and evaluated a total of 17 articles included in this analysis, achieving a coefficient of κ = 0.84. Results: The analysis revealed that the highest frequency of conducted studies was in the State of Mexico. The most common risk factors identified were environmental, pharmacological, consumption habits, and gynecological factors. Conclusions: Identifying the main risk factors for cleft lip and palate in the Mexican population will enable the implementation of preventive measures aimed at reducing exposure to these factors. Additionally, early intervention can improve the quality of life for individuals affected by this condition.

## 1. Introduction

Cleft lip and palate is the most frequent congenital malformation worldwide. This anomaly is produced by partial or complete fusion in the facial process during embryonic development. Both genetic variants and environmental etiologies may be involved in this failure, and it occurs with a prevalence of 1 per 1000 or 1500 births [1,2,3]. This congenital malformation was identified in the 4th century B.C [1]. The multifactorial nature of this condition makes prevention difficult and makes it challenging to determine a therapeutic target [4].

These clefts, along with other defects and syndromes that involve cleft lip and palate as one of their symptoms, amount to more than 400 different syndromes [5,6]. This is because of the chromosomal aberrations or monogenic diseases that can occur in 30% of cases [7].

Unlike non-syndromic cleft lip and palate, which is derived from the interaction between genetic and environmental factors, representing around 70% of cases, non-modifiable factors (sex, race, family history, etc.) and modifiable factors (diet, health status, medication, etc.) can exert an effect for a period of up to two months after conception [8].

Various studies carried out in recent years have identified several risk factors related to cleft lip and palate, including socio-economic factors, tobacco consumption, alcohol, drugs, maternal factors, infections by viruses and bacteria, genetic alterations and mutations, as well as occupational exposure [1,9,10,11].

However, the study of cleft lip and palate in the Mexican population is not applicable or insufficient to adequately characterize it. This is due to the frequency of risk factors, as well as their geographical distribution, which have been studied little.

Although the Ministry of Health in Mexico estimates a rate of 1 per 750 births, the prevalence of cleft lip and palate in this country is more complex and depends on the state in which the study was conducted [12,13].

Therefore, the purpose of this study was to carry out a systematic review to determine the frequency of risk factors associated with cleft lip and palate.

## 2. Material and Methods

### 2.1. Research Design

A descriptive and retrospective review of science articles published for the elaboration of this systematic review was carried out to resolve the question “What are the common risk factors associated with the development of cleft lip and palate in Mexican states?”.

### 2.2. Search Strategy

A search was conducted in the Scielo, Science Direct, PubMed, and EBSCO databases without restrictions based on the year of publication and applying language filters to English and Spanish. Key search terms included “cleft lip and palate” AND “risk factors” AND “epidemiology OR epidemiological”. The studies were screened for inclusion using predefined criteria. The selected items were organized into eight regions: northwest, northeast, west, east, northcentral, southcentral, southwest, and southeast. To enhance understanding of the factors, they were grouped into five categories: (1) socio-economic; (2) hereditary family history; (3) gynecological and perinatal; (4) habits and medicines; and (5) environmental factors.

### 2.3. Eligibility Criteria

The population, intervention, comparison, and outcome (PICO) synthesis tool was utilized to evaluate the eligibility criteria, which were defined as the following:

patient = patient diagnosed with cleft lip and palate in Mexico; **i**ntervention/exposure = systematic review and analysis of existing research to evaluate the cleft lip and palate associated with risk factors in Mexico; **c**omparison = evaluate and compare risk factors and epidemiological characteristics across different regions in Mexico; outcome = identification of risk factors and epidemiological characteristics associated with cleft lip and palate in different regions of Mexico.

*Inclusion criteria:* Studies with patients diagnosed with left clip and palate, without restriction with gender and age; studies that evaluated human participants; and studies conducted only in Mexican states. The studies evaluated included the following: (i) studies reporting patients diagnosed with cleft lip and palate; (ii) studies including maternal, paternal, and neonatal epidemiological characteristics associated with cleft lip and palate; (iii) prospective or retrospective studies; (iv) studies specifying the state or city within Mexico where they were conducted; and (v) studies reporting frequencies of risk factors associated with cleft lip and palate.

*Exclusion criteria:* Studies were excluded if they met the following criteria: (i) had inadequate information; (ii) included other countries or if they were studies which included Mexico and other countries; (iii) were studies which did not include risk factors included in the search strategy; (iv) studied cleft lip and palate associated with other syndromes; (v) were review articles, meta-analyses, letters to the editor, or original research articles that did not report on cleft lip and palate or were not directly related to the review’s purposes.

### 2.4. Data Extraction and Evaluation

Three independent authors (J.A.S.L., S.L.V., and N.M.F.) selected and evaluated studies that were deemed relevant for the complete text evaluation. After discussion and consensus among the three authors, the articles with pertinent content were chosen. A total of 20 articles were included in this analysis, achieving a coefficient of κ = 0.84. After selection of the articles, two authors (O.T.M and VH.T.R) individually extracted the quantitative and qualitative data from the selected articles. Standardized forms were used to facilitate the analysis of the information. The extracted information included the following data items: title, authors, year of publication, Mexican states in which data were collected, type of cleft present, number of participants including controls, as well as participants’ age range.

**Figure 1:**
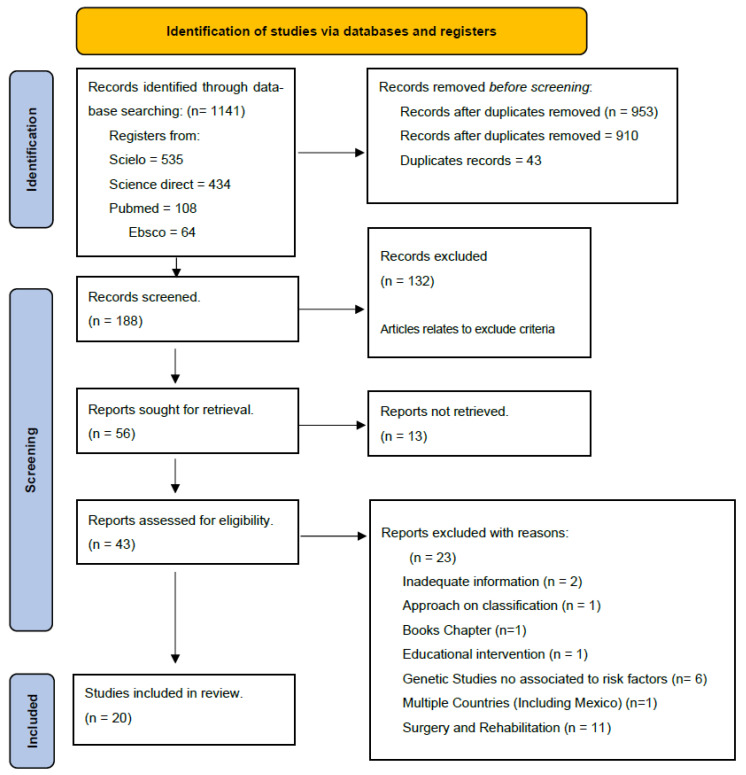
Four-phase PRISMA chart for searching and selecting items. PRISMA flow chart for the systematic review. Of the 1141 articles found in the 4 databases included in the search, 20 (1.75%) studies were selected for analysis. Following this, the articles underwent a title and abstract review, with the exception of any articles displayed.

A third reviewer (R.G.G.) was consulted in cases of disagreement between the two authors. The PRISMA guide was used for item selection according to inclusion and eligibility criteria. The term “Mexico” was used to exclude items pertaining to other countries. The review followed the PRISMA chart (Figure 1), with peer review of abstracts and full texts to verify compliance with the pre-established inclusion criteria.

Risk of bias was reviewed according to the Methodological Index for Non-Randomized Studies (MINORS), in which the global ideal score is 16 for non-comparative studies and 24 for comparative studies [14]. The data analyzed from each article were collected in Excel (Microsoft Excel 365, version 16.87, Microsoft 2024. Ciudad Santa Fe, MX) in the following order: study authors, year, place, presence or absence of cleft lip and palate, and risk factors associated with cleft lip and palate.

## 3. Results

**Figure 2:**
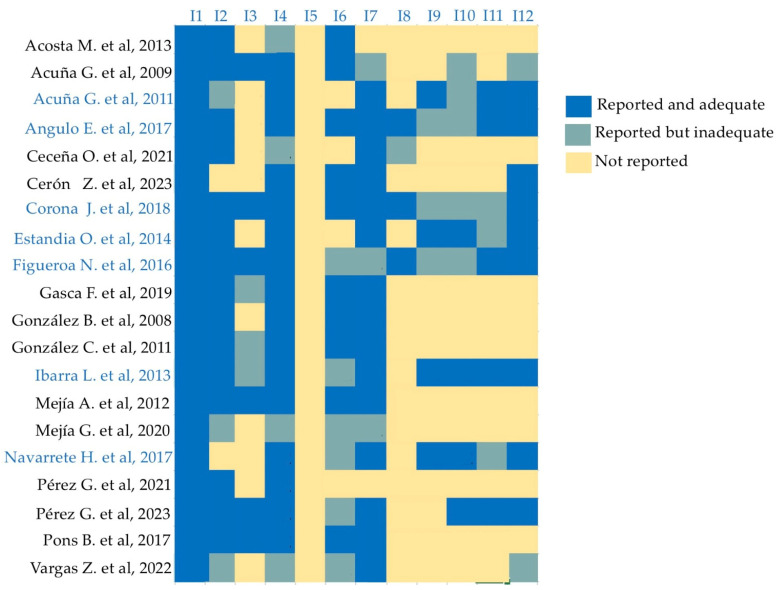
MINORS heat map. Items (I): I1—a clearly stated aim; I2—inclusion of consecutive patients; I3—prospective collection of data; I4—endpoints appropriate to the aim of the study; I5—unbiased assessment of the study endpoint; I6—follow-up period appropriate to the aim of the study; I7—loss to follow-up less than 5%; I8—prospective calculation; I9—an adequate control group; I10—contemporary groups; I11—baseline equivalence of groups; I12—adequate statistical analyses [9,13,15,16,17,18,19,20,21,22,23,24,25,26,27,28,29,30,31,32].

Out of the total of 1141 (100%) results in the databases, only 20 (1.75%) articles that studied risk factors associated with cleft lip and palate, published between the years 2003 and 2023, were included. According to the MINORS instrument (Figure 2), all studies had a clear aim (I1), the majority included consecutive patients (I2), had unambiguous explanation of the criteria used to evaluate the main outcome (I4), and included all patients in the study without a loss less than 5% (I7). The principal weaknesses (yellow) were prospective collection data (I3), the fact that blinding evaluation was absent in all, and the prospective calculation of the study. Because of the lack of case–control studies, I9, I10, and I11 showed more yellow spaces. The mean global score for non-comparative studies was 12, and it was 21 for comparative studies (Figure 2, references in blue). 

The states in which these studies were conducted were Baja California [15], Sinaloa [16], Nuevo León [17], Jalisco [18,19], Querétaro [20], Guerrero [21], Campeche [9,22], Puebla [23], Mexico State [24,25,26], and Mexico City [27,28,29,30]. Three of the studies were conducted throughout the entire republic [13,31,32]. The state of Mexico stands out among the 36,493 samples of cleft lip and palate patients with a total of 3174 cases, followed by the state of Jalisco, with 2008 cases, and Mexico City (CDMX), with 1244 cases accumulated over a period of 20 years (2003–2023). Similarly, the state of Colima has the lowest incidence of cases, accumulating only 21 cases in the same period.

**Figure 3:**
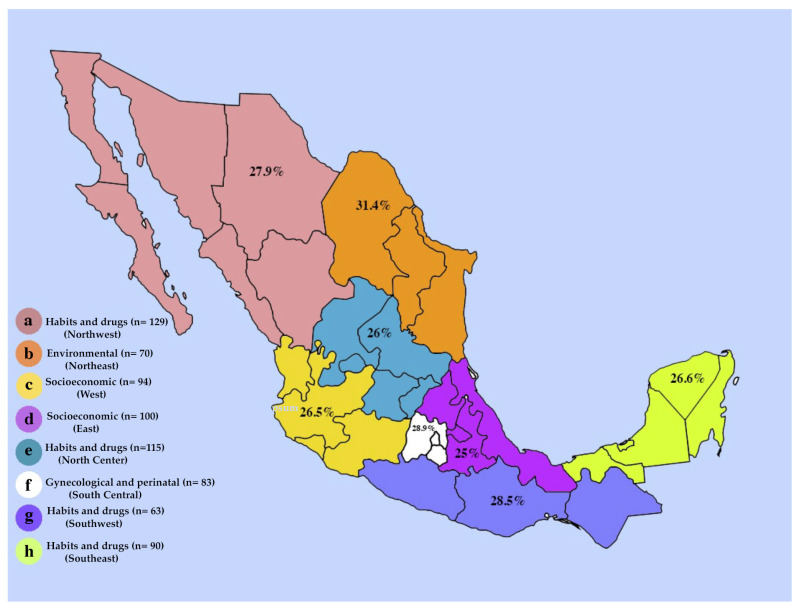
Map of the most common risk factor categories by region. (a) Lower California, Baja California, Sonora, Durango, Sinaloa; (b) Coahuila, Nuevo León, Tamaulipas; (c) Nayarit, Jalisco, Colima, Michoacán; (d) Hidalgo, Tlaxcala, Puebla, Veracruz; (e) Zacatecas, Aguascalientes, San Luis Potosí, Guanajuato, Querétaro; (f) State of Mexico, Mexico City, Morelos; (g) Guerrero, Oaxaca, Chiapas; (h) Tabasco, Campeche, Yucatán, Quintana Roo.

Furthermore, the categories of factors were charted according to the region of Mexico in which they were examined (Figure 3). We note that the most analyzed factors in the northwest region, as well as in the northcentral, southwest, and southeast regions, were those in the habits and medicines category. The northeast region has a higher frequency of environmental factors, as it was the region that assessed all of these factors. Studies conducted in the western and eastern regions found that socio-economic factors had a high frequency; the same was observed in the southcentral region with gynecological and perinatal factors. On the contrary, hereditary–family background factors showed low frequencies in all regions of the country. It is important to note that not all studies assessed all categories.

**Figure 4:**
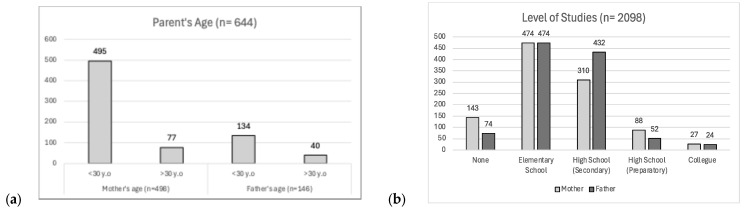
Socio-economic risk factors related to cleft lip and palate: (**a**) risk factors associated with parents’ age; (**b**) risk factors related to education level.

Next, in the category of socio-economic factors, risk factors were subsequently gathered that were studied in 14 of the 15 articles; among them, the following are highlighted: “mother’s age” in 9 articles, “father’s level of education” in 5 articles, and “fathers’ age” in 4 articles. The highest incidence of cleft lip and palate cases was found in mothers under the age of 30 (84.5%). The predominant age of mothers was in the range of 24–26 (26.1, SD ± 1.39, min 24.5, max 28) years, while the father’s age was 25–29 years (27.4, SD ± 2.7, min 23, max 30.3). The studies by Perez-González A [29,30] and Pons-Bonals A [20] divided mothers into age groups, with ages 20 to 29, 21 to 25, and 26 to 30 years showing the highest frequency. Pons-Bonals divided the father’s age into groups, and the 21- to 25-year-old group showed the highest frequency of cases [20] (Figure 4a). Parents who only completed primary school were classified as possessing a low level of education (45.18%). The percentage of mothers and fathers who completed senior high school was 4.19% and 2.48%, respectively. The percentage of parents who completed senior high school and university studies was low (1.29% and 1.14% for mothers and fathers, respectively). A total of 10.34% did not have any academic background (Figure 4b).

**Figure 5:**
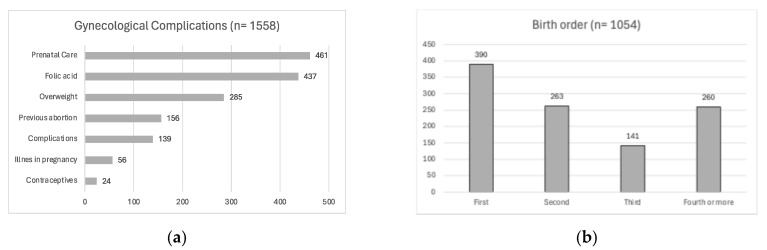
Gynecological and perinatal factors in Mexico: the category graphs depict the most common cases of (**a**) gynecological complications and (**b**) birth order.

In the category of gynecological and perinatal factors, 12 articles analyzed showed a total of 16 factors, with the “sex” factor being the most studied due to its presence in all of them. Meanwhile, six articles focused on the “prenatal care” factor, and seven articles discussed “folic acid consumption”. Folic acid use and prenatal care were prevalent in most cases. These factors were followed in terms of frequency by being overweight and having previously undergone an abortion (Figure 5a).

From the perinatal factors present in cases of cleft lip and palate, it was observed that, regarding the order of birth, it is highlighted that, in most cases, the patient is the firstborn of the family (Figure 5b). Regarding the sex factor, 34,092 cases were evaluated, of which 58% of the cases were male and 42% were female, a ratio of 1.4:1.

**Figure 6:**
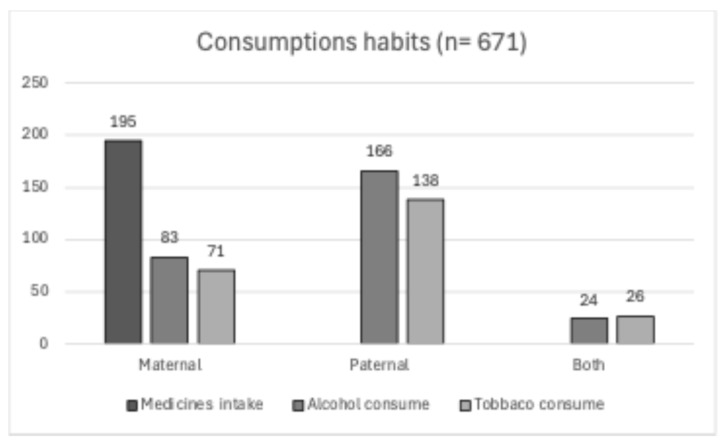
Frequencies of risky habits and medicine intake: fathers consumed alcohol and tobacco more frequently than mothers, and only a small percentage of both mothers and fathers used these substances simultaneously.

Similarly, the category of habits and drug factors gathers 8 risk factors, analyzed in 12 articles, highlighting the medicine intake factor with/without prescription as the most analyzed, as it was present in 9 articles, a frequency of 24.4%. However, regarding frequencies, the highest percentage was the alcohol factor in the father, present in 24.7% of cases, followed by 20.6% of cases that identified the tobacco factor in the father (Figure 6).

**Figure 7:**
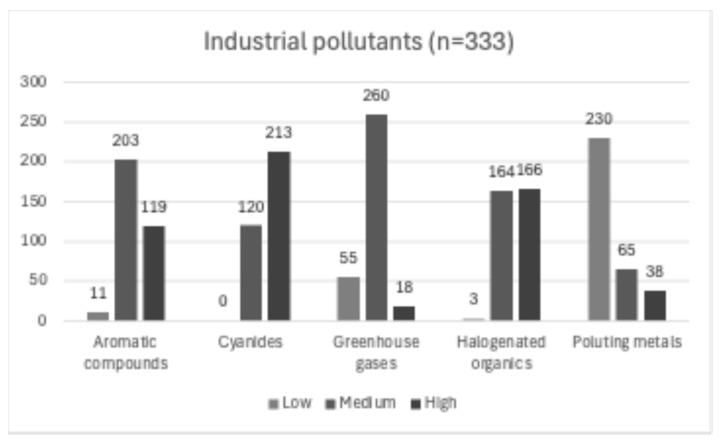
Frequency of cases related to industrial pollutant concentrations.

Industrial pollutants were observed in 333 cases (7 articles) that were presented within the areas of low, medium, and high concentrations of industrial pollutants, of which 63.9% of the cases were exposed to high concentrations of cyanides. For polluting metals, 69% of cases occurred in areas with low concentrations of metals. As for halogenated organic pollutants, a higher prevalence of cases was found in areas of high (49.8%) and medium (49.2%) concentrations, with a difference of only two cases between the two. A total of 60.9% of patients were exposed to medium concentrations of aromatic compounds, and 78% of cases were identified in areas with medium concentrations of greenhouse gases (Figure 7).

Finally, two works, in addition to reporting the frequency of some risk factors, evaluated the interaction of folic acid intake with polymorphisms in the methylenetetrahydrofolate (MTHFR) gene in the mestizo Mexican population. Ibarra-López et al., 2013 [21], found that mothers with 677CT or 677TT genotypes had a higher risk of having a child with cleft lip and palate, and in the case of the latter genotype, the risk increased with a lack of folate supplementation during the first trimester of pregnancy. Estandia-Ortega et al., 2014 [28], concluded that intake of folic acid and the TT genotype with the MTHFR C677T polymorphism in children independently reduced the risk of cleft lip and palate.

## 4. Discussion

On several occasions, the risk factors associated with lip and palate fissure and their inter-relationship with the socio-demographic, socio-economic, and pollution characteristics of different areas around the world have been studied in detail; however, most use information of Anglo-Saxon (European or North American) or Asian origin, which, due to racial and environmental differences, cannot be applicable or accurate for the Mexican population. In addition, the associated risk factors depend on the methodological design of the research.

Epidemiological studies suggest that maternal risk factors play an important role in the development of different birth defects where the parent’s conditions are not the same, according to data that are little studied and often unknown. It is noteworthy that most of the frequencies in this paper indicate only maternal characteristics, because studies are generally oriented to identify exposure to risk factors during pregnancy [9,15,16,20,25,26]. However, some authors consider that paternal influence can be exerted before conception through toxicant transmission in semen or even genetic mutations; e.g., paternal smoking could interfere with the genesis of the male gamete [10] or other epigenetic changes in the nucleic acids of spermatozoa could be produced by the influence of environmental factors [33]. For this reason, Nguyen et al., 2007, evaluated the occupation of parents of children with isolated oral clefts in Norway, a variable missing in the studies included [34].

In this review, throughout the investigations, it was found that within the category of socio-economic factors, the age of the mother exerted the strongest influence. Nevertheless, at present, its place in terms of association has been diminished, and it is increasingly less frequently analyzed. Surprisingly, a higher proportion of parents (father and mother) under the age of 30 was found in several manuscripts [9,15,18,20,24,26], as opposed to the findings of previous research, where the influence of age on the risk of cleft lip and palate increases with the aging of one of the parents and decreases if one of the parents is young [30,35]. Similar results were obtained in a Nigerian population [36].

As a result of our findings, we concur with the assertions of Corona R. et al. regarding the parents’ educational attainment: primary school education serves as the highest level of education in the majority of cases and consistently reflects a low socio-economic status [18].

Hereditary family history was recorded for most of the articles; however, it was not specified whether it was maternal or paternal history, leaving aside the branch of genealogy affected by this malformation. For this reason, little can be achieved in terms of prevention and genetic counseling. The data from the articles were collected from the areas of gynecology and obstetrics, which is why the focus granted to the paternal genealogy branch is minimal. Thus, in these cases, it is unknown whether the father had a child(s) with cleft lip and palate prior to the one identified in the current study [16,18,20,24,25,30]. A study by Muñoz et al. reported in 2001 that cases of cleft lip and palate are twice as common in families with a hereditary family history of malformations [6].

Undoubtedly, the consumption of folic acid is internationally recognized as a preventive measure for birth defects. In our results, we can observe that most of the mothers consumed folic acid and attended their prenatal care appointments; despite this, their son presented with cleft lip and palate. As set out in NOM-034-SSA2-201325, a daily intake of 4 mg of folic acid is recommended for pregnant women and their partners with a family or personal history of malformations or living in the geographical areas with the highest incidence of cases (Secretaria de Salud, 2023) [12]. Similarly, it is important to emphasize that this measure should be applied during the preconception period, which is three months before pregnancy and lasts until the 12th week of gestation. Thus, regularly, the mother starts consuming folic acid at the time when she is diagnosed with pregnancy; that is, when the pregnancy is advanced by a month or more and when the folic acid may no longer be as effective [9,26]. Interestingly, Ibarra et al., 2013, found an association between a lack of folic acid supplementation and the combination of the genotype variants 677TT and 677CT in the maternal MTHFR gene. Opposite results have been found in Norwegian [37] and French [38] populations. We did not find other genes evaluated in conjunction with risk factors in the Mexican population.

Body mass index (BMI) was only studied in mothers, and we can observe a clear association with cleft lip and palate and BMI, as most mothers were overweight or obese before, during, and after the pregnancy in question [16,18,26].

On the other hand, in our study, a higher frequency of cases was found for the firstborn child [20,24], contrary to what was stated by other authors, who found that the risk of cleft lip and palate increases when the number of offspring increases [9,16,19,26].

Tobacco and alcohol consumption are the factors that have the highest association with cleft lip and palate; however, these previous studies focused mainly on the mother, with few studies reporting this habit in both parents [9,20,27]. Nevertheless, these factors appeared with higher frequency in parents of children with cleft lip and palate, according to a study by Martinelli et al. in 2020 [11]. They claimed that the use of tobacco in men prior to insemination increases the risk of non-syndromic orofacial fissures [11].

In the same context, several authors pointed out that the pattern and severity of malformations caused by the use of medication depend on the dose, time, and duration of exposure, with the first trimester of pregnancy being the stage of highest risk. The risk of having a child with cleft lip and palate is five times higher when medications are consumed during pregnancy; our results were in agreement with this observation: this factor was found in more than half of the mothers [1,10,35,39].

Environmental risk factors were the least analyzed throughout the country; however, parents exposed to pollution were predisposed to having children with this malformation. Several authors found that increased exposure to solid urban waste raised the rate of cleft lip and palate, as did exposure to toxic substances in the environment and at work, such as wood smoke, chlorinated solvents, fertilizers, and pesticides [15,26,30,31]. While there was not a direct link between the two, Gasca et al. showed that there was a geographical proximity between cases of cleft lip and palate and exposure to environmental pollutants such as carbon dioxide, arsenic, mercury, nickel, lead, cadmium, and cyanide [17]. These pollutants are linked to birth defects and are found in moderate-to-high amounts in urban areas.

Importantly, this birth defect has a great deal of different causes; thus, it is difficult to determine a foolproof way to prevent it. This problem is related to a fusion of the palatine and labial processes that happen at a time during pregnancy when the mother usually does not know she is pregnant [11].

Regarding limitations, the differences in methods used to evaluate each variable studied complicate the homogeneity of results presented in this study. Another important limitation is related to the fact that the majority of the studies in this systematic review met the inclusion criteria but lacked evaluations of paternal risk factors. These risk factors, which include age, educational level, substance use, and exposure to industrial pollutants, could potentially be linked to the father’s occupational characteristics. However, the reviewed articles do not fully document this connection.

## 5. Conclusions

It is important to highlight that the parental influence is related to exposure to toxic agents associated with consumption and/or a polluted environment, including alcohol and tobacco consumption, in which the influence of the father stands out more. This influence may be linked to a higher risk of cleft lip and palate in newborns. This review of risk factors for cleft lip and palate in Mexico not only helps with early diagnosis but also enables preventive measures and improved prenatal care. Early intervention and timely treatment can significantly improve the quality of life for children born with cleft lip and palate, emphasizing the importance of ongoing research and increased efforts to reduce the impact of these risk factors and, ultimately, to improve the well-being of affected individuals and their families. In addition, it is crucial to emphasize that more research is needed in Mexico to evaluate contaminating risk factors. Finally, additional genetic studies of parents of children with cleft lip and palate must be carried out in order to gain a deeper understanding of the factors that influence this condition.

## Data Availability

No new data were created or analyzed in this study.
